# Chronic pain in older adults with psychiatric disorders: the DoCPPA study protocol

**DOI:** 10.1186/s12877-025-06239-3

**Published:** 2025-08-25

**Authors:** Hélène Saint-Martin, Isabelle Rouch, Bernard Laurent, Arlette Edjolo, Elodie Pongan, Mathieu Herrmann, Christelle Creac’h, Karine Pérès, Hélène Amieva, Jean-Michel Dorey

**Affiliations:** 1 Department of Aging Psychiatry, Le Vinatier Hospital Center, 95 Boulevard Pinel, Bron, 69500 France; 2 Bordeaux Population Health Center, INSERM, U1219, ACTIVE Team, University of Bordeaux, Bordeaux, France; 3 Memory Clinical and Research Center of Saint-Etienne, University Hospital of Saint-Etienne, Saint-Etienne, France; 4 Neuropain Team, Lyon Neuroscience Research Center, INSERM, U1028, CNRS, UMR 5292, Lyon, France; 5 Memory Clinical and Research Center of Lyon, Hospices Civils de Lyon, Charpennes Hospital, Villeurbanne, France

**Keywords:** Chronic pain, Psychiatry, Aging psychiatry, Older age, Elderly, Depression, Anxiety, Severe mental illness, Dementia, Epidemiology

## Abstract

**Background:**

Chronic pain (CP) and psychiatric disorders (PD) are common in older adults, and they both may significantly impact patients’ functioning and quality of life. However, research on the prevalence and impact of CP in people with PD remains limited– especially in older adults– and psychiatric care often neglects somatic comorbidities.

**Objectives:**

The main objective of the DoCPPA study is to determine the prevalence and characteristics of CP in older adults with PD followed-up in psychiatric services. Our secondary objective is to estimate associations between CP and various clinical indicators related to physical, cognitive, and mental health, as well as quality of life.

**Setting:**

Department of Aging Psychiatry of Le Vinatier Hospital Center (France, Bron), inpatient and outpatient psychiatric services.

**Method/design:**

Cross-sectional monocentric study with 430 patients with PD under psychiatric care. The inclusion period will be 36 months. The patients will be evaluated using validated scales and neuropsychological assessments.

**Discussion:**

This study aims to contribute to improving care in aging psychiatry, a field where a major challenge lies in the management of multiple chronic conditions.

## Introduction

### Conceptual framework

#### Chronic pain and psychiatric disorders in older adults

Chronic pain (CP) is defined as any persistent or recurrent pain lasting longer than 3 months [1]. Rheumatic and joint pathologies are the most common causes in adults, followed by cancer-related pain and neuropathic pain linked to chronic somatic disorders [2]. CP is very common [3, 4] and increases with age: the TNS Sofres study carried out in France on a sample of over 30,000 people found a CP prevalence of 20% in the under-35s, reaching 50% in the over-75s [3].

On the other hand, nearly one in two people over 65 report a history of psychiatric disorder (PD), and 17% of them currently suffer from at least one PD [5]. These include depression (a widespread disorder, present in up to 25% of geriatric inpatients), bipolarity, anxiety disorders (present in 14% of people over 65), psychotic disorders, and addictive disorders [5, 6]. As people age, PD may differ in their symptomatology and repercussions, and, therefore, in the way they are treated [6, 7].

#### Chronic pain and psychiatric disorders: a bidirectional relationship

Pain plays a key role in the emergence and perpetuation of psychiatric symptoms, with a bidirectional relationship. People with CP are indeed at greater risk of developing sleep disturbances, anxiety disorders, depressive symptoms, or suicidal ideation [8–12]. Unstabilized CP can even increase the risk of suicide [13] and up to 10% of people who died by suicide had reported or would have reported CP– although this proportion is likely underestimated [14]. Conversely, several longitudinal studies have shown that the existence of psychiatric vulnerability favors the incidence of CP [15], and the prevalence of CP is higher in patients with depression and bipolar disorders than in the general population [16, 17].

Although this higher prevalence is still poorly understood, one possible explanation may be that PD– and, in particular, severe conditions [18]– are often associated with earlier and more frequent somatic comorbidities as well as poorer medical care, for a variety of reasons (e.g., stigmatization, poor compliance, difficulty in seeking help, precariousness, etc.) [19]; and that these comorbidities are regular sources of CP. Anxiety and depressive symptoms have also been identified as obstructive factors in pain recovery, prompting healthcare professionals to detect and address them [20]. Several authors suggested that CP could, conversely, hinder the clinical recovery of psychiatric pathologies [17]; although empirical evidence is still lacking.

#### Impact on cognitive functioning and psychobehavioral symptoms

Beyond PD, CP also appears to be associated with cognitive and behavioral decompensation.

Recent longitudinal studies suggest that CP is associated with an increased risk of cognitive decline [21–23] and even dementia [23]– although the results on this latter point diverge [24]. CP could also potentiate the accelerated cognitive decline observed in certain severe PD, such as schizophrenia and bipolar disorders [25, 26]. On the other hand, cognitive disorders could favor the maintenance of pain and its chronicization; in particular executive and attentional deficits, preventing the crucial use of self-regulation strategies when confronting to stressors [27]. Thus, some authors discuss the concept of pain as a cognitive disorder [28].

CP may also be a risk factor for the onset or worsening of psychobehavioral symptoms, particularly in patients with neurocognitive disorders [29, 30]– and pain management would conversely reduce behavioral and affective symptoms [31, 32] in these patients.

#### Impact on quality of life and care pathways

Like many chronic diseases, both CP and PD have a major impact on quality of life, and significantly increase the need for care.

CP has been shown to affect the locomotor sphere, limit mobility, and increase the risk of confinement and social isolation [33, 34]. It is also associated with premature mortality and increased use of care services [35], particularly in older adults [36]. Concerning mental illness, life expectancy for people with PD is significantly lower than for the general population [37] and the leading causes of such premature deaths are due to physical health problems (e.g., diabetes; HIV).

In addition, the presence of multiple chronic disorders can exacerbate the impairment caused by each condition. Pain has thus been shown to increase disability and reduce quality of life in people with serious mental illnesses [38], such as mood [39] and psychotic disorders [40]; and psychiatric morbidity may adversely affect quality of sleep and quality of life of patients with CP [41]. Such findings have prompted health authorities to draw up recommendations for the management of polypathological patients, emphasizing their need for coordinated care. This particularly applies to older adults, as the prevalence of multiple concurrent chronic conditions increases with age. The Insee-Credes survey, carried out in France on over 20,000 people, showed that polypathology was the rule among older people: 93% of those aged 70 and over reported at least two illnesses, and 85% at least three [42]. For example, in a sample of older adults living in nursing homes, 13% had both CP and a severe depressive episode [43]. As the prevalence of multiple concurrent conditions increases, the risks of mortality, poor functional status, unnecessary hospitalizations, or adverse drug events also increase [44]. Care should therefore be structured to consider both CP and PD– especially in older patients–to prevent cascading decompensation, accelerated aging and reduced life expectancy.

### Problem identification, original contribution and review of the literature

From a public health perspective, joint management of different disorders could thus help optimize care and reduce healthcare costs. This represents a particular challenge in aging psychiatry, as the proportion of older patients admitted to psychiatric wards is already high (e.g., patients over 60 currently account for 25–38% of hospitalizations related to bipolar disorder) and expected to increase in the future [45–47]. Indeed, the life expectancy of patients with PD is better than in the past– though still shorter than in the general population [48]– and some estimates predict a doubling in the prevalence of chronic PD (such as schizophrenia or bipolar disorder) beyond the age of 65 [6].

However, neither research nor care dedicated to older adults with PD sufficiently addresses the issue of CP. Research on the prevalence of CP and its impact on the clinical expression of PD remains rare [49], especially in aging psychiatry. Furthermore, the care provided in aging psychiatry services is usually focused on psychiatric symptoms, paying little attention to associated comorbidities. Moreover, most of the studies on CP in older adults with PD have focused on individuals living in nursing homes, with the risk of confounding psychiatric diagnoses with neurocognitive disorders. For example, the prevalence of CP has been found to be between 50% and 80% in nursing home residents suffering from neurocognitive disorders, chronic psychiatric pathologies, or mood disorders [50]. To the best of our knowledge, there are no studies on CP in geriatric psychiatric patients outside the institutional setting, despite several studies confirming the value of this type of research [21, 51]. The latter highlight the research gaps regarding CP, both in the older population and in psychiatry, and outline prospects for progress in terms of pharmacological and non-drug management. They bring out the frequent exclusion of older patients and patients with severe PD from treatment efficacy studies (e.g., 60% of the 75 trials dedicated to pain psychotherapy in 2020 excluded people with severe mental illness). Yet, the lack of evidence for the efficacy of drug and non-drug treatments– and for their potential interactions with psychiatric symptomatology and psychiatric care– can either reduce their use in these patients (and diminish their chances of recovery) or lead to harmful side effects and reduced life expectancy. A better understanding of CP characteristics in patients with PD (particularly in older patients) would help to promote the implementation of systematic pain assessments in psychiatry, using suitable tools (e.g., taking non-verbal data into account). It would then encourage care professionals to use more appropriate medications, as many overlaps have been highlighted between psychotropic and analgesic medications [52, 53], and as pain treatment success rates can vary in psychiatric populations [54]. Onwumere et al. [51] also suggest that a variety of criteria should be taken into account when analyzing pain characteristics in this type of patients; among them, caregiver burden, functional status, and psychiatric diagnosis category have been considered in the development of our study.

The primary aim of our study is to gain a better view and understanding of (i) the number of persons affected by CP in aging psychiatry; (ii) the type and characteristics of pain syndromes presented by these patients; (iii) the type of analgesic medication and non-drug treatments they have had or currently have access to; and (iv) the impact of pain on their daily-life activities. Our secondary objectives concern the relationship between CP and other aspects of health and life: (i) psychiatric history, diagnosis, symptoms, and treatments; (ii) cognitive functioning; (iii) psycho-behavioral symptoms; (iv) metabolic conditions; (v) functional status; (vi) quality of life; and (vii) caregiver burden.

## Methods and analysis

### Study design

Cross-sectional descriptive monocentric non-comparative study.

### Sample size

Considering a power of 95%, a risk α of 0.05 and a CP expected prevalence of 50%, the number of subjects required is 385. Including a margin of around 10% to compensate for probable missing data, the number of subjects required is 430.

### Setting and patients

This study will focus on adult patients living in France, aged 65 and over, suffering from PD and under psychiatric care in the Department of Aging Psychiatry of Le Vinatier Hospital Center. Le Vinatier covers a population of almost 800,000, i.e. 50% of the population of the Rhône department. The Department of Aging Psychiatry cares for patients aged 65 and over who present either a late-onset PD; a new decompensation of an early-onset PD; or a neurocognitive disorder with psychiatric features.

Among them, 430 patients will be recruited over a 36-month period to form our sample: 230 to 250 inpatients and 180–200 outpatients.

Inclusion criteria are:

- Patients from the outpatient and inpatient services of the Department of Aging Psychiatry of Le Vinatier Hospital Center.
- 65 and over.
- Patients fluent in French.
- Patients affiliated to or entitled under a social security scheme.

Exclusion criteria are:

- Presence of somatic progressive or unstable disease (e.g., newly diagnosed cancer).
- Moderately severe or severe cognitive impairment defined by a MMSE (Mini-Mental Status examination) [55] score below 15/30.

### Study withdrawals

Patients withdraw from the study if they are unwilling or unable to continue for medical reasons. They will not be replaced.

### Primary variables

See Table 1 for a summary of the variables and corresponding criteria.

**Table 1:** Variables and corresponding criteria

Domain	Variables	Evaluation tools
Physical health	Chronic pain criteria	STOPNEP (first two questions)
	Chronic pain characterization	STOPNEP
	Sensitization syndrome	CSI-A
	Pain treatment	History of drug and non-drug analgesic treatment Current prescription: analgesic molecules and their dosage
	Impact of chronic pain on daily life activities	BPI-SF
	Cardiovascular risk factors	Hypertension, diabetes, smoking history, hypercholesterolemia, BMI
Mental health	Psychiatric and neurological diagnosis	ICD-10 diagnoses made by a professional psychiatrist
	Psychiatric symptoms	GAD-7 CES-D
	Psychiatric history and treatment	Age at onset for psychiatric and neurological disorders History of psychiatric hospitalizations and suicidal behaviors Current prescription: psychotropic molecules and their dosage
	Psychobehavioral symptoms	NPI
	Cognitive functioning	Cognitive efficiency: MMSE Memory: 5-Word Test; RL/RI-16; Digit Span subtest of the WAIS battery Executive and attentional functioning: FAB; GREFEX’s verbal fluency test and Trail Making test; Stroop Victoria; TAP; Coding subtest of the WAIS battery Social cognition: mini-SEA facial emotion recognition test; Pain recognition and pain intensity judgment tasks
Quality of life	Autonomy level	Type of housing (i.e., personal residence; retirement home; nursing home) Protective measures Human assistance IADL
	Health-related quality of life	SF-12
	Sleep	PSQI
	Caregiver burden	mini-Zarit

The presence and characterization of CP will be assessed using the Study of Prevalence of Neuropathic Pain questionnaire (STOPNEP), Central Sensitization Inventory– Part A (CSI-A), and Brief Pain Inventory– Short Form (BPI-SF). The STOPNEP questionnaire [3] comprises 10 questions among which only the first two questions (i.e., “At present, do you suffer from one or more pain(s) felt every day?” and “For how long have you suffered from pain(s) felt every day?“) will be administered to all patients to identify the presence of CP. Only those meeting CP criteria will be offered the rest of the questionnaire, as well as the CSI-A and BPI-SF. The third question in the STOPNEP questionnaire consists of localizing their pain from a list of body sites and, if they have mentioned several locations, reporting the single location of the most bothersome pain. The next five questions relate to the duration (less than 6 months; 6 to 12 months; 1 to 3 years; more than 3 years) and intensity (Likert scales ranging from 0–10) of the most bothersome pain. The patient is then asked two questions about the history of the disease (i.e., circumstances of pain onset and management). The CSI-A [56–59] comprises 25 questions rated on a 5-point Likert scale (0 to 4). Total score, increasing with the severity of the syndrome, ranges from 0-100; a score of 40 or more is in favor of a central sensitization syndrome. The second part of the BPI-SF [60, 61] comprises 10 questions. It will be used to assess the impact of CP on daily life on a scale from 0, “Does not interfere with”, to 10, “Interferes completely with”: general activity, mood, walking ability, normal work, relations with other people, sleep, enjoyment of life, self-care, recreational activities, and social activities.

Four questions will then be asked to every patient (with or without CP) in order to characterize pain assessment and management practices of the attending physician as well as the doctors and carers involved in the psychiatric follow-up.

Finally, analgesic medication prescription will be recorded (i.e., number and classes of molecules, dosage).

### Secondary variables

Regarding physical health, several cardiovascular risk factors will be listed (hypertension, diabetes, smoking history, hypercholesterolemia, Body Mass Index (BMI)).

Regarding the psychiatric and behavioral symptoms, the current diagnosis will be determined by the referring psychiatrist, who is currently managing the patient in consultation (outpatient department) or hospitalization (inpatient department), based on the International Classification of Diseases, 10th Revision (ICD-10). Anxiety symptoms will then be investigated using the Generalized Anxiety Disorder– 7 items (GAD-7) that comprises seven questions rated on a 4-point Likert scale (0 to 3) [62, 63]. It assesses the frequency of occurrence of seven symptoms of anxiety over the past 14 days. Total score ranges from 0 to 21; a score of seven or more is in favor of a general anxiety disorder. Depressive symptoms will be investigated using the Center for Epidemiologic Studies - Depression (CES-D) [64, 65] which comprises 20 questions rated on a 4-point Likert sale (0 to 3). Its purpose is to assess the frequency of occurrence of symptoms frequently associated with depression over the past seven days. Total score ranges from 0 to 60; a score of 16 or more is indicative of a depressive disorder. Psychobehavioral symptoms will be assessed using the Neuropsychiatric Inventory (NPI) [66, 67]. This inventory records neuropsychiatric symptoms observed by caregivers or healthcare professionals in 12 different domains: delusions, hallucinations, agitation, depression, anxiety, elation, apathy, disinhibition, irritability, aberrant motor behavior, and sleep and appetite disturbances. For each behavior, the symptoms frequency, severity and impact are assessed on Likert scales.

Finally, socio-demographic data will be collected from the patient: age, sex, socio-cultural level (i.e., number of years of education), living context (i.e., type of housing, human assistance). Health-related quality of life will be assessed using the Health Survey Short Form (SF-12) [68–70]: this 12-item questionnaire asks about the functional impact of physical and psychological health. Physical (PCS-12) and mental (MCS-12) quality of life sub-scores and total score are calculated using scoring algorithms [71]; the highest scores indicate a good quality of life. The Pittsburgh Sleep Quality Index (PSQI) [72, 73], a 19-question self-questionnaire, will be used to assess sleep in terms of quality, latency, duration, efficiency, disturbances, medication use, and impact on daytime activity. These seven component sub-scores and the total score (ranging from 0 to 21) will be calculated; the highest scores indicate poor sleep quality. Functional status will be estimated using the Instrumental Activities of Daily Living (IADL) [74, 75], an eight-item questionnaire measuring the patient’s level of dependence in activities of daily living (i.e., telephone use; errands; food preparation; housekeeping; laundry; means of transport; treatment; and money handling). Each item is scored 0 or 1, giving a total score of 0–8. Lower scores indicate a high level of dependence. Finally, if applicable, the mini-Zarit scale [76, 77] will be used to assess the caregiver burden, using 12 questions rated on a 3-point Likert-scale (0; 0.5; 1). Total score ranges from 0 to 7, the highest scores indicating greater caregiver burden.

Regarding the cognitive profile, the MMSE test [55] will be used to assess global cognitive functioning and eligibility criteria. The 5-Word Test [78] and the Frontal Assessment Battery (FAB) [79] will be used to assess verbal episodic memory and executive functioning respectively. In addition, a more complete evaluation will be administered to inpatients, including:

- The Free and Cued Selective Reminding Test (FCSRT) [80], designed to assess verbal episodic memory skills.
- The Digit Span subtest of the WAIS battery [81], which assesses short-term memory and working memory skills.
- GREFEX’s verbal fluency test and Trail Making Test (TMT) [82, 83] and Stroop Victoria [84], which assess executive functions (flexibility and inhibition).
- The Digit Symbol Substitution subtest of the WAIS battery [81] and the Test battery for Attentional Performance (TAP) [85] designed to assess the psychomotor speed and the attentional sphere.
- The facial emotion recognition test of the mini-Social cognition & Emotional Assessment (mini-SEA) [86] and the pain recognition and pain intensity judgment tasks based on the work of Ceresetti et al. [87] assessing the socio-cognitive sphere.

### Data collection procedure

At the first visit, the psychiatrist in charge of the patient will present the study and ensure that the patient meets the inclusion criteria. The patient will be given a two-day cooling-off period, after which he/she expresses non-opposition if he/she wishes to participate in the study and is informed of the date of the next visits.

The assessment may take one or two visits, depending on the patient’s status: one for outpatients; two for inpatients, to carry out additional cognitive assessments. If applicable, the patient’s caregiver will be invited at the end of a visit to complete the mini-Zarit and the NPI. If not applicable, the NPI will be completed by a professional caregiver.

### Bias mitigation measures

Various measures will be taken to reduce selection bias:

- Systematic recruitment: every patient seen for a consultation or hospitalized during the inclusion period will be invited to take part in the study.
- Inclusion criteria: few exclusion criteria, in order to constitute a sample as representative of the population as possible.
- Benefit/risk balance: relatively short evaluation, encouraging participants to adhere.

Other measures are taken to reduce information bias:

- Evaluation criteria: scales validated in French and in our population.
- Participants will be blinded to the study assumptions to avoid information bias. They will receive the explanations at the end of the session.
- Data collection: assessment by a healthcare professional trained in psychometrics and not involved in the patient’s routine care, to limit interpretation bias.

### Data analysis

The prevalence study will be based on data concerning the presence and type of CP.

Inferential statistical analyses will then be performed to study cross-sectional associations. Parametric analyses will be carried out, after verification of the required application criteria (normality of distribution, homogeneity of variance, sphericity for repeated measures). If these criteria are not met, appropriate non-parametric tests will be carried out. The following statistical analyses are envisaged:

- Univariate ANOVAs and correlation studies to study associations between the presence and characteristics of CP and the various clinical indicators related to health and quality of life.
- Multivariate analyses, including multiple linear and logistic regression models, to account for different covariates (e.g., age, gender, level of education, comorbidities, analgesics, psychotropic drugs).

As the prevalence of CP varies with age, the analysis will be controlled for age. In addition, further analysis will be considered by stratifying the population into different age subgroups, as the impact of CP, its management and its association with PD may vary significantly with age.

## Discussion

This research will add important insights to the rare literature on the prevalence and impact of CP in older adults with PD. The provided data will enable us to assess various hypotheses concerning CP and its relationships to other aspects of health.

### Chronic pain in older adults with psychiatric conditions

Firstly, we hypothesize a high prevalence of CP in older adults with PD [15], even higher than in the general population. In particular, we expect to find a very high prevalence of CP in older adults with mood disorders [16, 17, 51].

Concerning the type of pain, we will list the most commonly affected body sites and we expect back pain to be the commonest type of pain presented, as in the general population [88, 89]. The investigation of the history of the disease and previous diagnoses and the CSI-A scores will help us estimate the frequency of primary and secondary pain syndromes [90].

We also hypothesize a high impact on daily-life activities. We expect this impact to be correlated to the pain intensity, duration, and number of body sites affected by pain [91]. We will examine which of the various activities listed in the BPI-SF are most affected.

Regarding pain management, we will look for markers of poor pain management (e.g., patients who do not report having been assessed for pain; whose pain has not been diagnosed; or who have no treatments), as pain appears to be regularly under-detected, under-assessed and under-treated [52], particularly in PD patients [40]. We expect to observe this even more in older PD patients, for whom polypathology and polymedication is frequent and difficult to deal with. Regarding treatment history, we expect the number of different treatments (drug and non-drug) taken to date to correlate with the duration of symptoms– a marker of therapeutic wandering. We will look for the number of CP patients who do not [52] have a combination of drug and non-drug treatment, despite recommendations. Finally, we will examine the frequency of prescriptions with multiple side effects in older adults, particularly focusing on anticholinergic and sedative effects.

### Cross-sectional associations

Secondly, by comparing patients with CP to those without, we will seek to identify the clinical variables that are impacted by the presence of CP, concerning health (physical, psychic, cognitive) and quality of life. Indeed, we hypothesize that CP patients have more cardiovascular risk pathologies [92] and that the Drug Burden Index is higher [93, 94]. We also hypothesize that CP patients present more intense psychiatric [95–97] and behavioral symptoms [29, 30]; have suffered from psychiatric conditions for a longer time [39]; are more likely to have had suicidal behaviors [14, 98]; and have prescription of psychotropic drugs including more molecules with more regular changes [99]. Regarding cognition, we hypothesize that CP patients have more cognitive disorders, particularly in the attentional and executive spheres [27, 28]; and are more likely to have been diagnosed with a neurocognitive disorder [23]. Finally, we hypothesize that CP patients have reduced functional autonomy in activities of daily living [39, 51, 100] - this could mean that they are more often institutionalized, need more home help, and are more often under protective measures. We also hypothesize that they have a poorer quality of life [40, 41, 51]; more sleep disorders [41]; and their caregiver burden is greater [101–103].

The use of multivariate analyses will enable to account for the relative weight of each of these variables on the health and quality of life of older patients with PD, and to identify the most significant risk and protective factors (e.g., combinations of disorders that are most deleterious to quality of life). We will also look for mediating factors between CP and other variables. For example, cognitive inhibition deficits and impulsivity could mediate the relationship between CP and suicidal behavior [98, 104].

### Perspectives

This study is of great interest from a scientific, clinical, and public health point of view.

As we mentioned earlier, research studies on the prevalence, impact, and treatment of pain in psychiatry are still lacking. With this study, we hope to improve scientific knowledge on the prevalence and characteristics of CP in older adults with PD. Numerous studies have demonstrated that certain mental health problems can contribute to the emergence, aggravation, or perpetuation of pain, and vice versa. Nevertheless, most studies have chosen either a dimensional (symptom assessment) or a categorical (diagnostic) approach. One of the strengths of our study lies in its dual approach to these disorders, combining a record of diagnoses made during the course of care with an instantaneous assessment of the symptoms presented by the patient. Furthermore, to the best of our knowledge, this will be the first study to focus on older adults under psychiatric care– and not just those in nursing homes– to look at the full range of disorders presented by older adults. Using standardized and validated scales will allow to compare our results with those obtained in other populations. The sample size, made possible by the scale of the hospital services involved, also represents a major statistical advantage.

From a clinical point of view, CP appears to be under-assessed and under-treated in this fragile population. We hope that the identification of the impact of CP on clinical characteristics will help to promote the introduction of systematized pain assessments in routine practice and to improve pain management throughout the psychiatric care pathway, by promoting coordination between the various care providers (psychiatrists, algologists, pharmacists, etc.).

By focusing on the central issue of pain, we hope to be able to identify new ways of improving the health, functional status, and quality of life of older adults with PD. From a public health standpoint, this improvement could potentially lead to a reduction of their use of the healthcare system through, for example, shorter and less frequent psychiatric hospitalizations; optimization of drug prescriptions; reduced need for home help; and lightening of caregivers’ burden. By promoting pain management in psychiatric wards, we hope to provide better care coordination for people with multiple pathologies and reduce their recourse to parallel care channels (notably geriatric care). This issue is particularly relevant given the current shortage of general practitioners, especially in certain regions.

Subsequently, cross-sectional associations will enable to support the implementation of a longitudinal study, designed to determine the nature of these associations.

### Limitations

Firstly, the structuring of care at Le Vinatier Hospital Center may lead to selection bias. By default, and to ensure continuity of care, adults with chronic PD continue to be managed in the adult psychiatric department they belong to as they age, as long as they continue to require psychiatric care. Only after a break in care of at least five years are these people, now over 65, transferred to the Department of Aging Psychiatry. As adult departments are not involved in this research, this type of chronic patients will not be represented in our sample. Involving additional care providers from the psychiatric, geriatric, or neurological fields in our future works would enable to capture a larger, more representative sample of older adults with moderate to severe mental disorders.

Secondly, some of our evaluation methods may introduce measurement bias. Regarding pain assessment: since the evaluation does not include a physical examination, we will have difficulty in pinpointing the etiology of pain and, in particular, identifying patients with primary pain. This would have represented a significant advantage, as pain etiology is rarely investigated in large studies of prevalence in older people [53]. The Central Sensitization Inventory [56, 58] was chosen for this purpose, as it is the first tool to assess central sensitization. Although it has not been validated in older and/or psychiatric populations, the CSI-A score, when considered alongside other criteria (e.g., pain chronicity and distribution), in accordance with the latest international recommendations [90], will assist us in identifying patients with primary pain. Regarding facial expressions recognition tasks: the pain recognition and the pain intensity judgment tasks, developed with Simon et al. [105, 106] (Simon D, Craig KD, Miltner WHR, Rainville P. (in press). Brain responses to dynamic facial expressions of pain. Pain. Unpublished.) material and based on Ceresetti et al. [87] work, have not been validated. We shall therefore confine our analyses to detecting differences in the ability to recognize facial expressions related to intra-sample variations (e.g., presence or absence of CP).

## Data Availability

No datasets were generated or analysed during the current study.
